# Effect of Creatine Monohydrate Supplementation on Various Hematological and Serum Biochemical Parameters of Male Albino Mice following Neonatal Hypoxia-Ischemia Encephalopathy

**DOI:** 10.1155/2013/286075

**Published:** 2013-09-19

**Authors:** Shahid Iqbal, Nabia Nazir, Quratulane Gillani, Atif Akbar, Furhan Iqbal

**Affiliations:** ^1^Zoology Division, Department of Zoology, Institute of Pure and Applied Biology, Bahauddin Zakariya University, Multan 60800, Pakistan; ^2^Department of Statistics, Bahauddin Zakariya University, Multan 60800, Pakistan

## Abstract

*Background*. Present study was designed to report the effect of 2% creatine monohydrate supplementation for 8, 12 and 15 weeks on hematology and serum biochemical profile of male albino mouse following hypoxic ischemic insult on postnatal day 10. *Methods*. 66 Blood samples (2% creatine monohydrate supplemented (*N* = 34) and unsupplemented (*N* = 32)) were analyzed for various hematological (blood glucose, packed cell volume, total WBC count, total RBC count) and serum biochemical parameters (cholesterol, AST, ALT, HDL, LDL, total protein, triglycerides). *Results*. ALT had higher concentrations in mice feeding on normal diet for 8 (*P* > 0.01) and 12 weeks (*P* > 0.01) following asphyxia and in 12 weeks treatment without asphyxia (*P* = 0.006) when compared with the creatine supplemented mice. LDL (*P* = 0.011) and cholesterol (*P* > 0.01) had higher concentrations in mice on normal diet for 12 weeks following hypoxia ischemia. Cholesterol (*P* > 0.01) in 12 and glucose (*P* = 0.006) in 15 week treatment group had significantly lower concentrations in creatine supplemented male albino mice when compared with untreated group following hxpoic-ischemic insult. *Conclusion*. We concluded that creatine supplementation following hypoxic ischemic insult helps in maintain the normal blood chemistry.

## 1. Introduction

Hypoxic ischemic encephalopathy (HIE) is a common cause of neonatal brain injury with an incidence rate of 2 in 1000 term infants in the developed world. 15–20% of infants with HIE die in neonatal period, and 25–30% of survivors are left with permanent neurodevelopmental abnormalities [1]. Hypoxic ischemic injury in neonates is caused by variety of conditions such as birth asphyxia, cardiac arrest, or respiratory failure. Lack of regenerative ability in the central nervous system after injury is considered to be its fundamental cause [2]. HIE can lead to postneonatal neurological sequelae, that is, cerebral palsy, mental retardation, learning disability, and epilepsy [3–6]. Data from Pakistan has shown that it is one of the leading causes for admission in a neonatal unit [7]. It has also been shown that most causes of neonatal morbidity in Pakistan are preventable [8].

Creatine (Cr) and phosphocreatine (PCr) play an essential role in the storage and transmission of phosphate bound energy [9]. Creatine is an amino acid derivative that is found naturally, to small extent, in human body [10]. Liver, pancreas, and kidney synthesize approximately 2% of the total body creatine while 98% of creatine is taken with food. Creatine is naturally present in meat and to some extent in green leaved vegetables. 60% of creatine found in the body is in the form of creatine phosphate [11]. Creatine has been shown to be neuroprotective in various neurological conditions [12]. Acute oral creatine supplementation reduces mental fatigue on a serial calculation task [13]. Creatine shows protective effects in models of acute neuronal damage, such as traumatic brain injury [14] and stroke [15]. In animal models of neurodegenerative disease, creatine monohydrate supplementation has been shown to improve brain functioning, reduce oxidative stress, and attenuate neuronal degeneration [16–18]. Despite of extensive studies on creatine and its neuroprotective effects, little information is available regarding the effect of creatine monohydrate supplementation on blood chemistry following hypoxic ischemic encephalopathy.

Present study was conducted to study the effect of 2% creatine monohydrate supplemented or normal rodent diet on selective parameters of albino mice blood profile under normal conditions as well as following neonatal hypoxic ischemic encephalopathy (HIE) at postnatal day 10.

## 2. Materials and Methods

### 2.1. Experimental Animals

Male albino mice were used as experimental animals and maintained in cages filled with wood chips at animal facility, Institute of Pure and Applied Biology (IPAB), Bahauddin Zakariya University, Multan, Pakistan. In breeding colony, standard mouse diet and water were available. Room temperature was maintained at 22 ± 1°C. The light/dark rhythm was maintained at 14 : 10 hours. The room was illuminated with artificial light at an intensity of about 200 W from 8 a.m. to 7 p.m. All the experimental protocols and mouse handling procedures were approved by the ethical committee of IPAB at Bahauddin Zakariya University Multan.

### 2.2. Murine Model of Hypoxic-Ischemic Encephalopathy

On postnatal day 10, corresponding to a brain development of full-term (40 weeks) gestational period in human fetus, male pups were anesthetized using isoflurane (3%) inhalation. A right lateral neck incision was made, and the right common carotid artery was ligated using polypropylene falcon USP 6 suture. Pups were kept on a hot plate with constant 36°C temperature during the surgery. The entire surgical procedure was completed within 10 minutes. Mice were then placed for 25 minutes in a hypoxic chamber with constant flow of 8% oxygen balanced with nitrogen. The hypoxic chamber was kept in water bath to maintain the ambient temperature inside the chamber at 36°C. Following hypoxic exposure, pups were returned back to their mothers for recovery.

### 2.3. Experimental Design

On the 18–20 days of life, experimental animals were separated from their parents and divided into four experimental groups. In first experiment mice were feed with diet supplemented with 2% creatine monohydrate, while control group were fed with standard rodent diet for 8 weeks following hypoxic ischemic insult on postnatal day 10. In second and third experimental series, the same design was followed as mentioned above expect that the duration of 2% creatine monohydrate supplementation and standard diet supplementation was 12 and 15 weeks, respectively, after weaning. A separate fourth cohort of animals was used to study the effect of 2% creatine monohydrate supplementation for 12 weeks on hematology and serum biochemistry of male albino mouse without hypoxic ischemic insult.

### 2.4. Blood and Serum Collection

At the end of each experiment, mice were anesthetized with 3% isoflurane, and blood was sampled either from retro-orbital sinus or through direct cardiac puncture. Blood was divided into two parts; one for the study of hematological parameters and second for serum biochemical profiling.

### 2.5. Hematological and Serum Biochemical Analysis

Hematological parameters (blood glucose level, mean corpuscular volume, packed cell volume, total red, and white blood cell count) and serum biochemical parameters (cholesterol, aspartate transaminase (AST), alanine transaminase (ALT), high density lipoprotein (HDL), low density lipoprotein (LDL), total protein, and triglycerides) were determined in treated and untreated male albino mouse blood samples by using Hitachi 902 automatic analyzer (Japan).

### 2.6. Statistical Analysis

All the data is expressed as mean ± standard deviation. Statistical package Minitab (version 13, Pennsylvania) was used for the analysis of results. Two-sample *t*-test was applied to compare various parameters of hematology and serum biochemical profile of albino mouse between 2% creatine monohydrate treated and their respective untreated controls following hypoxia ischemia encephalopathy. Same statistical test was applied to compare various studied parameters between 2% creatine monohydrate treated and untreated male albino mouse.

## 3. Results

### 3.1. Effect of 8 Week-Creatine Monohydrate Supplementation on Blood Profile of Albino Mouse after HIE

Results indicate that all other studied parameters remained unaffected when compared with 2% creatine monohydrate treated and untreated albino mice after 8 weeks of supplementation except ALAT concentration which was significantly higher (*P* > 0.01) in creatine untreated group (Table 1).

### 3.2. Effect of 12 Week-Creatine Monohydrate Supplementation on Blood Profile of Albino Mouse after HIE

When hematological and serum biochemical parameters were compared between mice supplemented with 2% creatine monohydrate for 12 weeks and their untreated control after HIE. It was observed that Cholesterol (*P* > 0.01), ALAT (*P* > 0.01) and LDL (*P* = 0.01) concentrations were significantly different in blood of creatine monohydrate treated and untreated albino mice (Table 2).

### 3.3. Effect of 15 Week-Creatine Monohydrate Supplementation on Blood Profile of Albino Mouse after HIE

Glucose (*P* = 0.006) concentration was found significantly different in blood of 2% creatine monohydrate treated and untreated albino mice after 15 weeks of supplementation (Table 3).

### 3.4. Effect of 12 Week-Creatine Monohydrate Supplementation on Blood Profile of Albino Mouse without HIE

LDL (*P* = 0.006) and ALAT (*P* = 0.006) concentrations were significantly different in blood of male albino mouse treated with 2% creatine monohydrate for 12 weeks when compared with creatine untreated male albino mice (Table 4).

## 4. Discussion

The decreased tissue oxygen supply below the normal level is called hypoxia while reduced blood flow to organ when cerebral artery is blocked by a clot is called ischemia. During hypoxia, reactive oxygen specie (ROS) formation is increased and leads to stress oxidative condition [19]. Blood is an important medium in assessing the health [20], and physiological and pathological conditions of animals can be assessed by the evaluation of hematological and biochemical analyses of the blood [21]. The present study was designed to evaluate the effect of 2% creatine monohydrate supplementation on the blood chemistry and hematology of albino mouse under hypoxic ischemic and nonhypoxic ischemic conditions.

Our results indicated that creatine supplementation after hypoxic and ischemic insult has no effect on studied hematological parameters of blood, as there was no significant difference in hematological parameters when compared with 2% creatine monohydrate supplemented and untreated control group after 8, 12, and 15 weeks of 2% creatine monohydrate supplementation (Tables 1, 2, and 3). Similar results were obtained in nonhypoxic ischemic group when hematological parameters were compared in male albino mouse on normal rodent diet with mouse supplemented with 2% creatine monohydrate for 12 weeks (Table 4). Our results are in agreement with Eijnde et al. [22] who had reported that all values of blood hematology in human males following creatine supplementation remained within the normal clinical range throughout the study with no significant difference.

In 2% creatine monohydrate supplemented albino mouse, for 15 weeks, after hypoxic ischemic insult, blood glucose level was lower (177.8 ± 18.4) as compared with the creatine untreated control group (211 ± 21), and the difference was statistically significant (*P* = 0.006) (Table 3). Decreased glucose level after creatine supplementation was possibly due to the fact that creatine supplementation leads to increased insulin production confirming the findings of Rooney et al. [23] who had reported that chronic supplementation of creatine leads to hyper secretion of insulin decreasing the blood glucose concentrations.

Our results revealed that mouse exposed to hypoxic-ischemic insult had high serum cholesterol level when compared with non-hypoxic ischemic group. Our results are consistent with the results of Li et al. [24, 25] who had reported that hypoxia is one of the key mechanism which leads to the hyperlipidemia. Similar results were also documented by Savransky et al. [26] as they observed that hypoxia raises serum cholesterol and LDL-C levels in mice fed on a regular diet. In creatine supplemented hypoxic-ischemic mouse blood cholesterol level was lower (358.4 ± 32.4) as compared with normal diet fed controls (753.3 ± 33.1), and this difference was statistically highly significant (*P* = *P* > 0.01) (Table 2). Earnest et al. [27] reported effect of Cr supplementation on the plasma lipid profile in human males and females and observed that individuals with high basal cholesterol levels exhibited a reduction in blood total cholesterol following creatine supplementation.

Our results indicated that mouse that suffers from hypoxia and ischemia had high ALAT level as compared with their uninsulted control group. Increased ALAT level after hypoxia was also reported by Savransky et al. [26] while investigating the effects of hypoxia on the liver in the absence of obesity. Statistically significant difference in ALAT level was observed in hypoxic ischemic mouse after 8 weeks (*P* = *P* > 0.01) (Table 1) and 12 weeks (*P* = *P* > 0.01) (Table 2) of creatine supplementation as compared with their normal diet fed mouse. ALAT variation was also observed in the group without hypoxia and ischemia, In creatine supplemented mouse ALAT level was lower (55.33 ± 5.03) as compared with untreated control group (81.00 ± 3.61), and this result was statistically significant (*P* = 0.006) (Table 4) indicating that creatine supplementation improves the liver functioning in albino mouse.

## 5. Conclusion

Our results indicated that creatine supplementation after hypoxic and ischemic insult has no effect on studied hematological parameters of blood while serum biochemical profile is significantly affected by hypoxia ischemia, and in creatine monohydrate treated groups, studied parameters had concentrations lower than untreated groups indicating that creatine supplementation maintains serum profile as higher values of certain enzymes like ALAT are indicators of abnormal physiology.

## Figures and Tables

**Table 1:** Comparison of various hematological and serum biochemical parameters between 2% creatine monohydrate treated and untreated (control) male albino mice for 8 weeks following hypoxia and ischemia: data is expressed as mean ± standard deviation.

Parameters	Creatine monohydrate treated group		Untreated control group		*P* value
	Mean ± SD	Range	Mean ± SD	Range	
Hematological profile					
WBC (×10^9^/L)	6.006 ± 2.648	11.5–2.65	5.833 ± 3.099	11.3–2.5	0.901 ns
RBC (×10^12^/L)	4.96 ± 2.62	1.39–10.11	5.28 ± 1.14	4.0–7.18	0.748 ns
PCV%	25.8 ± 11.1	11.3–38	27.53 ± 8.27	12.2–42.5	0.731 ns
Glucose (mg/dL)	161.1 ± 38.4	98–214	136.2 ± 19.1	101–156	0.142 ns
MCV (fp)	84.6 ± 85.4	29.6–258.2	53.0 ± 15.4	27.8–78.2	0.370 ns
Serum biochemical profile					
Total protein (g/dL)	38.00 ± 9.17	28–46	53.00 ± 1.00	53-54	0.106 ns
Triglycerides (mg/dL)	234 ± 125	130–393	280 ± 140	201–410	0.646 ns
Cholesterol (mg/dL)	824 ± 196	489–984	579 ± 208	411–811	0.174 ns
HDL (mg/dL)	49.00 ± 3.00	46–52	49 ± 1.00	48–50	1.000 ns
LDL (mg/dL)	662 ± 237	411–883	482 ± 222	283–722	0.408 ns
ALAT (*μ*/L)	23.67 ± 9.07	14–32	248.67 ± 8.08	240–256	*P* > 0.001***
ASAT (u/L)	156.0 ± 16.4	138–170	142.0 ± 20.0	122–162	0.417 ns

*P* < 0.05 = non-significant (ns), ***P > 0.001 = highly significant.

**Table 2:** Comparison of various hematological and serum biochemical parameters between 2% creatine monohydrate treated and untreated (control) male albino mice for 12 weeks following hypoxia and ischemia: data is expressed as mean ± standard deviation.

Parameters	Creatine monohydrate treated group		Untreated control group		*P* value
	Mean ± SD	Range	Mean ± SD	Range	
Hematological profile					
WBC (×10^9^/L)	5.91 ± 2.85	3.4–10.7	9.170 ± 6.518	5.15–20.5	0.347 ns
RBC (×10^12^/L)	6.79 ± 2.74	4.59–10.86	6.76 ± 2.60	3.95–9.61	0.985 ns
PCV%	27.83 ± 7.41	19.2–39.5	25.03 ± 5.42	20–33	0.492 ns
Glucose (mg/dL)	169.5 ± 78.8	101–280	115.8 ± 40.2	60–162	0.188 ns
MCV (fp)	47.1 ± 22.8	19.8–80.4	40.3 ± 11.9	26–50.6	0.544 ns
Serum biochemical profile					
Total protein (g/dL)	3.2 ± 0.1	3.1–3.3	34.7 ± 27.3	17.4–66.1	0.184 ns
Triglycerides (mg/dL)	427 ± 314	65–616	305.3 ± 68.0	228–356	0.579 ns
Cholesterol (mg/dL)	358.4 ± 32.4	318–394	753.3 ± 33.1	722–788	*P* > 0.001***
HDL (mg/dL)	20.67 ± 4.04	17–25	35.0 ± 20.0	18–57	0.347 ns
LDL (mg/dL)	240.3 ± 72.5	173–317	656.7 ± 21.9	632–674	0.011*
ALAT (*μ*/L)	72.33 ± 6.81	67–80	191.7 ± 10.4	180–200	*P* > 0.001***
ASAT (u/L)	231.7 ± 10.4	22–240	161.7 ± 22.5	140–185	0.639 ns

*P* < 0.05 = non-significant (ns), *P > 0.05 = Least significant, ***P > 0.001 = highly significant.

**Table 3:** Comparison of various hematological and serum biochemical parameters between 2% creatine monohydrate treated and untreated (control) male albino mice for 15 weeks following hypoxia and ischemia: data is expressed as mean ± standard deviation.

Parameters	Creatine monohydrate treated Group		Untreated control group		*P* value
	Mean ± SD	Range	Mean ± SD	Range	
Hematological profile					
WBC (×10^9^/L)	9.94 ± 5.64	3.2–17.4	9.94 ± 1.66	7.4–11.8	0.806 ns
RBC (×10^12^/L)	7.512 ± 0.963	6.25–9.03	7.55 ± 2.98	2.77–10.58	0.977 ns
PCV%	31.5 ± 13.1	13.5–52.3	29.11 ± 9.66	14.2–41.6	0.681 ns
Glucose (mg/dL)	177.8 ± 18.4	150–205	211 ± 21	176–245	0.006**
MCV (fp)	42.5 ± 17.2	15–71.6	53.9 ± 47.9	13.4–150	0.570 ns
Serum biochemical profile					
Total protein (g/dL)	3.83 ± 0.93	2.5–5	2.80 ± 1.66	1.6–4.7	0.423 ns
Triglycerides (mg/dL)	210 ± 146	59–399	331 ± 79.2	240–399	0.188 ns
Cholesterol (mg/dL)	178 ± 131	75–399	315 ± 192	129–513	0.356 ns
HDL (mg/dL)	31.00 ± 3.00	28–34	28.00 ± 8.00	20–36	0.605 ns
LDL (mg/dL)	69.7 ± 46.0	25–117	221 ± 169	60–397	0.110 ns
ASAT (*μ*/L)	227.7 ± 60.1	162–280	608 ± 330	144–889	0.110 ns
ALAT (*μ*/L)	40.8 ± 30.6	20–86	100.5 ± 76.2	42–208	0.242 ns

*P* < 0.05 = non-significant (ns), **P > 0.01 = significant.

**Table 4:** Comparison of various hematological and serum biochemical parameters between 2% creatine monohydrate treated and untreated (control) male albino mice for 12 weeks: data is expressed as mean ± standard deviation.

Parameters	Creatine monohydrate treated group (*N* = 10)		Untreated control group (*N* = 10)		*P* value
	Mean ± SD	Range	Mean ± SD	Range	
Hematological profile					
WBC (×10^9^/L)	4.3 ± 1.5	2.5–8.5	8.3 ± 2.6	4.6–13.3	0.302 ns
RBC (×10^12^/L)	5.69 ± 3.14	2.9–13.8	5.85 ± 2.67	2.1–10.5	0.903 ns
PCV%	25.13 ± 5.58	17.3–34.8	28.0 ± 10.1	11.4–45.5	0.467 ns
Glucose (mg/dL)	146.4 ± 23.2	107–193	156.1 ± 60.5	78–289	0.659 ns
MCV (fp)	55.1 ± 27.3	12.3–97.2	61.0 ± 45.3	20.1–150	0.740 ns
Serum biochemical profile					
Total protein (g/dL)	5.27 ± 1.23	3.9–6.3	40.9 ± 27.9	12.5–78.8	0.084 ns
Triglycerides (mg/dL)	226.3 ± 16.2	216–245	395.0 ± 93.8	334–503	0.092 ns
Cholesterol (mg/dL)	376 ± 283	176–791	765 ± 118	629–839	0.068 ns
HDL (mg/dL)	35.75 ± 9.22	23–45	20.67 ± 2.08	19–23	0.051 ns
LDL (mg/dL)	152.6 ± 64.7	735–742	666 ± 107	94–222	0.006**
ALAT (*μ*/L)	55.33 ± 5.03	50–60	81.00 ± 3.61	78–85	0.006**
ASAT (u/L)	215.7 ± 46.7	165–257	157.33 ± 6.35	150–161	0.165 ns

*P* < 0.05 = non-significant (ns), **P > 0.01 = significant.
